# The Effects of Anti-Dementia and Nootropic Treatments on the Mortality of Patients with Dementia: A Population-Based Cohort Study in Taiwan

**DOI:** 10.1371/journal.pone.0130993

**Published:** 2015-06-22

**Authors:** Chen-Yi Wu, Hsiao-Yun Hu, Lok-Hi Chow, Yiing-Jenq Chou, Nicole Huang, Pei-Ning Wang, Chung-Pin Li

**Affiliations:** 1 Department of Dermatology, Taipei Veterans General Hospital, Taipei, Taiwan; 2 Institute of Public Health & Department of Public Health, National Yang-Ming University, Taipei, Taiwan; 3 Department of Education and Research, Taipei City Hospital, Taipei, Taiwan; 4 Department of Anesthesiology, Taipei Veterans General Hospital, Taipei, Taiwan; 5 Department of Anesthesiology, School of Medicine, National Yang-Ming University, Taipei, Taiwan; 6 Department of Anesthesiology, National Defense Medical Center, Taipei, Taiwan; 7 Institute of Hospital and Health Care Administration, National Yang-Ming University, Taipei, Taiwan; 8 Department of Neurology, Taipei Veterans General Hospital, Taipei, Taiwan; 9 Aging and Health Research Center, National Yang Ming University, Taipei, Taiwan; 10 Brain Research Center, National Yang-Ming University, Taipei, Taiwan; 11 Division of Gastroenterology, Department of Medicine, Taipei Veterans General Hospital, Taipei, Taiwan; 12 National Yang-Ming University School of Medicine, Taipei, Taiwan; University Of São Paulo, BRAZIL

## Abstract

**Background:**

Few studies have examined the contribution of treatment on the mortality of dementia based on a population-based study.

**Objective:**

To investigate the effects of anti-dementia and nootropic treatments on the mortality of dementia using a population-based cohort study.

**Methods:**

12,193 incident dementia patients were found from 2000 to 2010. Their data were compared with 12,193 age- and sex-matched non-dementia controls that were randomly selected from the same database. Dementia was classified into vascular (VaD) and degenerative dementia. Mortality incidence and hazard ratios (HRs) were calculated.

**Results:**

The median survival time was 3.39 years (95% confidence interval [CI]: 2.88–3.79) for VaD without medication, 6.62 years (95% CI: 6.24–7.21) for VaD with nootropics, 3.01 years (95% CI: 2.85–3.21) for degenerative dementia without medication, 8.11 years (95% CI: 6.30–8.55) for degenerative dementia with anti-dementia medication, 6.00 years (95% CI: 5.73–6.17) for degenerative dementia with nootropics, and 9.03 years (95% CI: 8.02–9.87) for degenerative dementia with both anti-dementia and nootropic medications. Compared to the non-dementia group, the HRs among individuals with degenerative dementia were 2.69 (95% CI: 2.55–2.83) without medication, 1.46 (95% CI: 1.39–1.54) with nootropics, 1.05 (95% CI: 0.82–1.34) with anti-dementia medication, and 0.92 (95% CI: 0.80–1.05) with both nootropic and anti-dementia medications. VaD with nootropics had a lower mortality (HR: 1.25, 95% CI: 1.15–1.37) than VaD without medication (HR: 2.46, 95% CI: 2.22–2.72).

**Conclusion:**

Pharmacological treatments have beneficial effects for patients with dementia in prolonging their survival.

## Introduction

Around 24.3 million people suffer from dementia worldwide, and this number is expected to increase to 81.1 million by 2040 [1]. This increase in the prevalence of dementia raises questions about survival rates in patients with dementia. Accurate estimates of prognosis are critical for making decisions about treatment. This is also an important public health issue and provides information for policy makers and health care providers.

People with dementia reportedly have decreased survival compared to those without [2–7]. Clinical experience and empirical data suggest that dementia is a leading cause of death and shortens the lifespan of elderly patients [8]. However, limited information exists about the disease course and outcomes. Therefore, strategies to delay the progression of dementia and mortality are currently being investigated.

The efficacies of anti-dementia drugs, cholinesterase inhibitors, and memantine have been demonstrated in randomized controlled trials [9, 10]. Long-term observational controlled studies show that persistent anti-dementia drug treatment is associated with a slower decline in cognition, daily function, and global severity [11]. Nootropics are known as cognition enhancers that improve mental function and can be used to treat a range of conditions including memory and concentration problems [12–14]. However, limited information regarding the contribution of anti-dementia and nootropic treatments on the mortality of patients with dementia.

Under the health insurance policy in Taiwan, patients are reimbursed for anti-dementia medication, i.e., donepezil, rivastigmine, galantamine, or memantine, only if they are undergoing treatment for Alzheimer disease (AD) but not vascular dementia (VaD), while all dementia patients are reimbursed for nootropics. Nootropics and/or anti-dementia treatments were further compared in the degenerative dementia group, whereas for the VaD group, only nootropics were analyzed. Using the National Health Insurance Research database, we conducted a nationwide population-based cohort study to investigate the effects of long-term therapeutic effects of anti-dementia and nootropic medications on the mortality of patients with dementia between 2000 and 2010.

## Materials and Methods

### Data sources

The National Health Insurance (NHI) is a mandatory universal health insurance program offering comprehensive medical care coverage to all Taiwanese residents. Of the residents in Taiwan, 96% have joined the NHI program since 1996. The NHI sample files, which are constructed and managed by the National Health Research Institute (NHRI), consist of comprehensive utilization and enrollment information for a randomly selected sample of 1,000,000 NHI beneficiaries, representing approximately 5% of all enrollees in Taiwan in 2000. A multistage, stratified, systematic sampling design was used. There were no statistically significant differences in age or gender between the sample groups and all enrollees. The comprehensive health care data include the enrollment files; the claims data for outpatients, inpatients, and emergency room use; and the registry for drug prescriptions.

### Incident cases of dementia

We conducted a retrospective cohort study from January 1, 2000 to December 31, 2010, based on the 1,000,000 NHI sample files. All patients aged 65 years or older with incident dementia were included in our study.

We enrolled the incident cases of dementia by choosing those free of dementia diagnoses in 1999 before being enrolled, and the onset date of dementia defined according to the first appearance of the code in the claim data. A total of 12,193 patients with dementia were included in this study. Owing to the strict regulation regarding reimbursement of anti-dementia medication and complexity of the reimbursement procedure in Taiwan, a limited number of dementia patients received reimbursement for anti-dementia medications. In our study, AD patients were defined according to the International Classification of Diseases, Ninth Revision (ICD-9: 331.0), or depending on whether they ever received reimbursement for anti-dementia medication. Only 1,793 patients (14.7% of all dementia patients) were identified as having AD. Most patients (7,607, 62.4% of all dementia patients) with dementia were diagnosed with senile dementia (ICD-9: 290.0, 290.1, 290.2, 290.3). To present this valuable information regarding dementia patients, we chose to group these patients with non-vascular dementia under degenerative dementia. All patients with dementia were classified into VaD (ICD-9: 290.4) and degenerative dementia (i.e., all other dementia cases except VaD, i.e., ICD-9: 290.0, 290.1, 290.2, 290.3, 294.1, 294.2, 331.0, 331.1, and 331.2) in this study. We included the cases where the subjects were diagnosed with dementia at least three times at an outpatient clinic, or at least one time at admission.

### Control group

Subjects without dementia records were used as the control group. One control was selected to match each dementia patient by random sampling stratified for age and sex from the database within the same observational period. A total of 12,193 subjects served as a comparison group.

### Outcome variables

All study subjects were followed from the index date to withdrawal from insurance (mostly due to deaths at this age group), or December 31, 2010, which ever date came first. Subjects with the latter two conditions were considered censored in the analysis.

### Covariates

Baseline covariates included socioeconomic status (low, medium, high), urbanity (urban, suburban, rural), and the Charlson comorbidity index (CCI). Urbanity was included to minimize the potential confounding effects of differential accessibility to and availability of medical care. Since the NHI program is financed by wage-based premiums for people with clearly-defined monthly wages and by fixed premiums for people without clearly-defined monthly wages, the socioeconomic status was classified into three categories according to each patient’s own insurance wage [15]. The CCI, which is a weighted summary measure of clinically important concomitant diseases adapted for use with ICD-9-CM-coded administrative data, was used to control for comorbidities [16]. To calculate the index, we retrieved the underlying diseases from each study subject’s inpatient claims from 2000 to 2010.

### Anti-dementia and nootropic medications

We defined anti-dementia and nootropic medications according to the Anatomical Therapeutic Chemical Classification System. The usage of nootropics, including piracetam, ginkgo folium, dihydroergocristine, and dihydroergotamine, was recorded in the claim data. There were no strict reimbursement regulations for nootropic prescriptions among patients with dementia.

However, to apply for any anti-dementia medication, i.e., donepezil, rivastigmine, galantamine, or memantine, in-charge neurologists or psychiatrists are requested to provide information including at least one report of computed tomography, magnetic resonance imaging, or Hachinski ischemic score, and a thorough laboratory examination report and detailed medical summary are needed for confirming the diagnosis of AD. The patient’s scores on the Mini-Mental State Examination (MMSE) or Clinical Dementia Rating (CDR) are used to indicate their current level of cognitive function. The diagnosis of AD and approval of these anti-dementia medications are reviewed and confirmed by a board-certified neurologist or psychiatrist. Only patients with MMSE scores of 10–26 or CDR scores of 1–2 are allowed to be reimbursed for cholinesterase inhibitors, while patients with MMSE scores of 10–14 or CDR scores of 2 are allowed to be reimbursed for memantine. The patients’ conditions must be re-evaluated every year after the initial prescription, and they are asked to discontinue the use of these medications if their MMSE scores decline by more than 2 points, or if the CDR scores deteriorate. Donepezil and rivastigmine have been covered by health insurance since October 1, 2001, galantamine since January 1, 2003, and memantine since June 1, 2006. Rivastigmine has been covered by health insurance for the treatment of Parkinson’s disease with dementia since 2010.

### Statistical analysis

The demographic information of patients with or without dementia was compared using χ^2^ tests for categorical variables and *t*-tests for continuous variables. The mortality incidence was calculated by dividing the number of individuals who died by person-years at risk during the follow up. Survival curves and median survival times were constructed based on the Kaplan-Meier methods. Comparisons of survival rates were performed using log rank tests. The log-log survival curve was used to test the proportional assumption. The plots of log-log survival curves were not parallel, and the linear association with the log of time suggested the appropriateness of a parametric survival model with Weibull distribution. Two models were used to estimate the hazard ratios (HRs), with the basic model adjusted for age and sex, and the full model adjusted for age, sex, socioeconomic status, urbanity, and comorbidity.

We used SAS 9.1 (SAS Institute Inc., Cary, NC, USA) to link the data, and Stata 12 (Stata Corporation, College Station, TX, USA) to perform the statistical analyses.

### Ethical Approval

Insurance reimbursement claims adopted in this study were from Taiwan’s NHIRDs, which is available for research purposes. All information that would allow a specific individual patient to be identified was encrypted. The confidentiality of the data abides by the data regulations of the National Health Insurance Administration, Ministry of Health and Welfare, Taiwan. The study was in accordance with the Helsinki Declaration and was approved by the NHRI and the Institutional Review Board of Taipei City Hospital (IRB: TCHIRB-1021224-E).

## Results

The baseline characteristics of the individuals who participated in the study are presented in Table 1. A total of 24,386 patients (12,193 dementia cases and 12,193 non-dementia cases) were included and followed for an average of 4.56 years (93,481 person-years at risk with 9,548 deaths). The mean age for patients with incident dementia was 79.1 years (standard deviation [SD]: 7.1). The individual matching process between patients with dementia and control subjects resulted in comparable distributions of age and sex.

**Table 1:** Baseline characteristics of the study samples.

Variables	Dementia cases		Non-dementia cases		P
	(n = 12,193)	%	(n = 12,193)	%	
Age (mean ± SD)	79.1 ± 7.1		79.1 ± 6.9		0.55
65–69	1,194	9.79	1,194	9.79	1.00
70–74	2,145	17.59	2,145	17.59	
75–79	3,006	24.65	3,006	24.65	
80–84	3,057	25.07	3,057	25.07	
≥85	2,791	22.89	2,791	22.89	
Sex					
Male	5,934	48.67	5,934	48.67	1.00
Female	6,259	51.33	6,259	51.33	
Socioeconomic status					
Low	9,177	75.26	9,282	76.13	0.29
Medium	1,449	11.88	1,391	11.41	
High	1,567	12.85	1,520	12.47	
Urbanity					
Urban	6,288	51.57	6,174	50.64	0.35
Suburban	3,988	32.71	4,063	33.33	
Rural	1,917	15.72	1,955	16.04	
Charlson comorbidity index (mean ± SD)	1.93 ± 2.36		1.40 ± 2.33		<0.001
Myocardial infarction	450	3.69	427	3.50	0.43
Congestive heart failure	1,913	15.69	1,359	11.15	<0.001
Peripheral vascular disease	356	2.92	232	1.90	<0.001
Cerebrovascular disease	2,326	19.08	1,207	9.90	<0.001
Dementia	2,415	19.81	0		<0.001
Chronic pulmonary disease	2,566	21.04	1,627	13.34	<0.001
Rheumatologic disease	61	0.50	55	0.45	0.58
Peptic ulcer disease	1,841	15.10	1,318	10.81	<0.001
Mild liver disease	499	4.09	428	3.51	0.02
Diabetes (mild to moderate)	2,671	21.91	1,666	13.66	<0.001
Diabetes with chronic complications	1,118	9.17	601	4.93	<0.001
Hemiplegia or paraplegia	406	3.33	189	1.55	<0.001
Renal disease	871	7.14	621	5.09	<0.001
Any malignancy	1,139	9.34	1,404	11.51	<0.001
Moderate or severe liver disease	134	1.10	115	0.94	0.23
Metastatic solid tumor	354	2.90	558	4.58	<0.001
AIDS	0		0		

SD: standard deviation; AIDS: acquired immune deficiency syndrome

There were no differences in socioeconomic status or urbanity between the dementia and non-dementia groups. The average CCI score of the non-dementia group was 1.40 (SD: 2.33), while the average CCI score of the dementia group was 1.93 (SD: 2.36), with significantly higher incidences of congestive heart failure, peripheral vascular disease, cerebrovascular disease, chronic pulmonary diseases, peptic ulcer diseases, mild liver disease, diabetes, hemiplegia or paraplegia, and renal disease, but lower incidences of malignancy and metastatic solid tumors.

Patients with VaD were slightly younger in age than those with degenerative dementia (78.2 ± 6.7 for VaD, and 79.3 ± 7.1 for degenerative dementia, P < 0.001), and had a higher CCI (2.44 ± 2.55 for VaD, and 1.83 ± 2.30 for degenerative dementia, P < 0.001) (Table 2). Compared to female patients, male patients had a higher ratio of VaD, but a lower ratio of degenerative dementia (54.6% vs. 47.4%, P < 0.001).

**Table 2:** Crude mortality incidence rate ratios for the dementia and non-dementia group.

Variables	Dementia			No dementia
	Total	Vascular dementia	Degenerative dementia	
	(n = 12,193)	(n = 2,142, 17.6%)	(n = 10,051, 82.4%)	(n = 12,193)
Age (mean ± SD)	79.1 ± 7.1	78.2 ± 6.7	79.3 ± 7.1	79.1 ± 6.9
Male: Female	5,934:6,259	1169:973	4765:5286	5,934:6,259
Charlson comorbidity index (CCI)	1.93 ± 2.36	2.44 ± 2.55	1.83 ± 2.30	1.40 ± 2.33
CCI except dementia	1.74 ± 2.25	2.13 ± 2.40	1.65 ± 2.21	1.40 ± 2.33
Median follow-up, years (IQR)	2.72 (1.20–4.94)	3.03 (1.47–5.35)	2.66 (1.16–4.86)	3.93 (1.95–6.00)
Person-years at risk	40,788	7,755	33,033	52,693
Death	5,688	1,008	4,680	3,860
Mortality incidence (/1000 py)	139	130	142	73
Crude IRR (ref: non-dementia) (95% CI)	1.90 (1.83–1.98)	1.77 (1.65–1.90)	1.93 (1.85–2.02)	
Median survival, year (95% CI)				
Total	5.07 (4.91–5.25)	5.50 (5.05–5.94)	4.99 (4.83–5.18)	9.23 (8.90–9.44)
Male	4.36 (4.14–4.58)	4.88 (4.42–5.40)	4.22 (3.98–4.44)	8.55 (8.02–8.96)
Female	5.89 (5.65–6.13)	6.31 (5.66–6.95)	5.77 (5.54–6.08)	9.84 (9.39–10.89)
65–69 years	9.97 (9.22–10.32)	7.74 (6.81–8,91)	10.25 (9.97–—)	11.00 (11.00–—-)
70–74 years	7.55 (7.07–8.28)	7.64 (6.44–8.36)	7.55 (7.03–8.41)	>11
75–79 years	5.89 (5.48–6.26)	6.27 (5.43–7.11)	5.79 (5.41–6.16)	10.78 (10.17–—)
80–84 years	4.38 (4.14–4.67)	4.01 (3.53–4.73)	4.47 (4.20–4.79)	7.29 (6.89–7.60)
≥85 years	2.90 (2.76–3.11)	3.92 (3.21–4.51)	2.80 (2.64–2.98)	4.51 (4.25–4.78)

SD: standard deviation; CCI: Charlson comorbidity index; IQR: interquartile range; py: person-years; IRR: incidence rate ratio; CI: confidence interval

### Incidence rate ratio (IRR) of mortality for degenerative dementia and VaD

At the end of the study, 46.6% of the people with dementia died versus 31.7% of the people without dementia. The crude mortality incidence ratios and median survival stratified by sex and age among the dementia and non-dementia groups are shown in Table 2. Compared to the non-dementia group, the crude IRR of mortality among patients with dementia was 1.90 (95% confidence interval [CI]: 1.83–1.98). For patients with VaD the IRR was 1.77 (95% CI: 1.65–1.90), while the IRR for patients with degenerative dementia was 1.93 (95% CI: 1.85–2.02). Female patients had longer survival times than male patients, regardless of whether they were in the VaD, degenerative dementia, or non-dementia group. Compared to degenerative dementia, VaD had a shorter median survival time at ages 65–69 (7.74 years [95% CI: 6.81–8.91] for VaD vs. 10.25 years [95% CI: 9.97–-] for degenerative dementia), and the risk reversed in patients aged 85 years or over, where degenerative dementia had a shorter survival time (3.92 years [95% CI: 3.21–4.51] for VaD vs. 2.80 years [95% CI: 2.64–2.98] for degenerative dementia).

### IRR of mortality for dementia with and without medication

The crude mortality incidence ratios with detailed classifications of patients with VaD and degenerative dementia according to their usage of medications for dementia are presented in Table 3. Among subjects with degenerative dementia, those using nootropic and/or anti-dementia medications were younger than those who were not using medication. Compared to the non-dementia group, the IRRs of mortality among patients with degenerative dementia were 3.07 (95% CI: 2.91–3.23) for patients not using medication, 1.56 (95% CI: 1.48–1.65) for those using nootropics, 1.02 (95% CI: 0.79–1.31) for those using anti-dementia medication, and 0.80 (95% CI: 0.69–0.91) for those using both nootropic and anti-dementia medications. In patients with VaD, those using nootropics were younger than those who were not using this medication. The patients with VaD who were using nootropics had lower mortality (IRR: 1.41, 95% CI: 1.29–1.54) than patients with VaD who were not using this medication (IRR: 2.82, 95% CI: 2.54–3.12).

**Table 3:** Crude mortality incidence rate ratio among the vascular dementia and non-vascular dementia groups.

Variables	Vascular dementia (n = 2,142)		Degenerative dementia (n = 10,051)			
	No medication	Nootropics	No medication	Nootropics	Anti-dementia medication	Both medications
	(n = 750, 35.0%))	(n = 1,392, 65.0%)	(n = 4,130, 41.1%)	(n = 4,850, 48.3%)	(n = 240, 2.4%)	(n = 831, 8.3%)
Age (mean ± SD)	78.8 ± 6.8	77.9 ± 6.7	80.5 ± 7.4	78.8 ± 6.9	78.4 ± 6.3	76.7 ± 6.2
Charlson comorbidity index (CCI)	2.29 ± 2.49	2.52 ± 2.58	1.77 .77.58	1.93 .93.58	1.38 ±.38 5	1.60 ±.60 5
CCI except dementia	2.01 ± 2.34	2.20 ± 2.42	1.62 .62424	1.76 .76424	1.14 ±.1495	1.30 ±.30 5
Prescription cumulative days (IQR)						
Nootropics	0	210 (63–577)	0	140 (44–420)	0.00	175 (58–511)
Anti-dementia medication	0	0	0	0	308 (90–739)	336 (126–805)
Median follow-up, years (IQR)	2.04 (0.86–3.80)	3.66 (1.91–6.03)	1.79 (0.68–3.62)	3.25 (1.57–5.41)	3.04 (1.50–5.11)	4.13 (2.35–6.37)
Person-years at risk	2,009	5,746	10,333	18,118	853	3729
Death	415	593	2,321	2,077	64	218
Mortality incidence (/1000 py)	207	103	225	115	75	58
Crude IRR (ref: non-dementia) (95% CI)	2.82 (2.54–3.12)	1.41 (1.29–1.54)	3.07 (2.91–3.23)	1.56 (1.48–1.65)	1.02 (0.79–1.31)	0.80 (0.69–0.91)

SD: standard deviation; IQR: interquartile range; py: person-year; IRR: incidence rate ratio; CI: confidence interval

### Survival time for degenerative dementia and VaD

**Fig 1:**
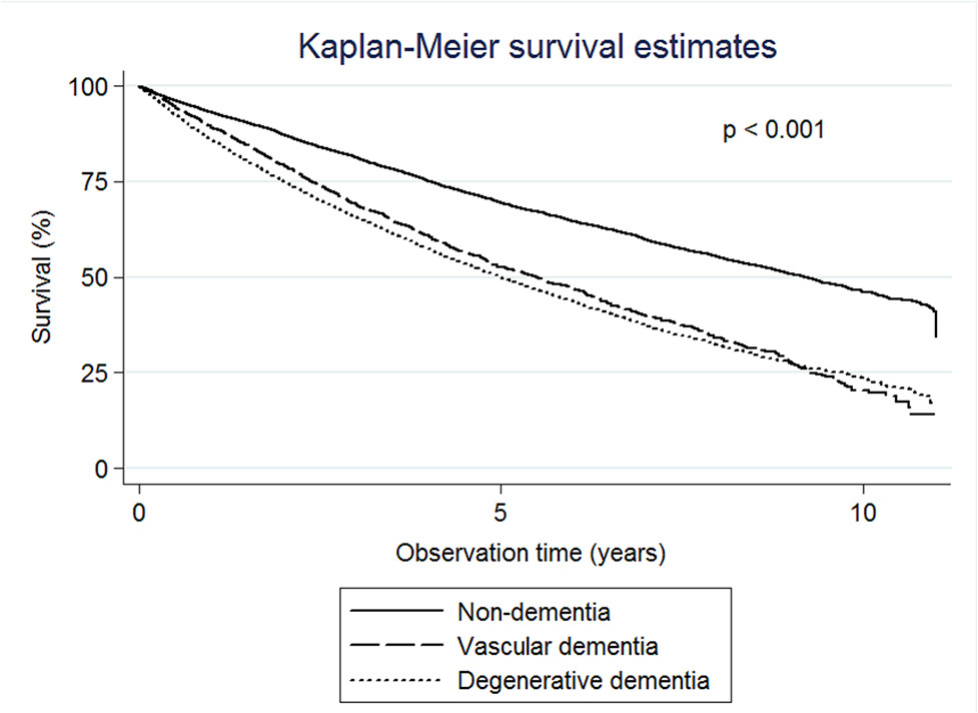
Kaplan-Meier survival estimates. The median survival times were 9.23 years for the non-dementia group, 5.50 years for vascular dementia, and 4.99 years for degenerative dementia.

The Kaplan-Meier analysis demonstrated a reduction in the survival probability associated with the different dementia groups compared to the non-dementia group. The median survival times were 9.23 years (95% CI: 8.90–9.44) for the non-dementia group, 5.50 years (95% CI: 5.05–5.94) for VaD, and 4.99 years (95% CI: 4.83–5.18) for degenerative dementia (Fig 1).

### Survival time for dementia with and without medication

**Fig 2:**
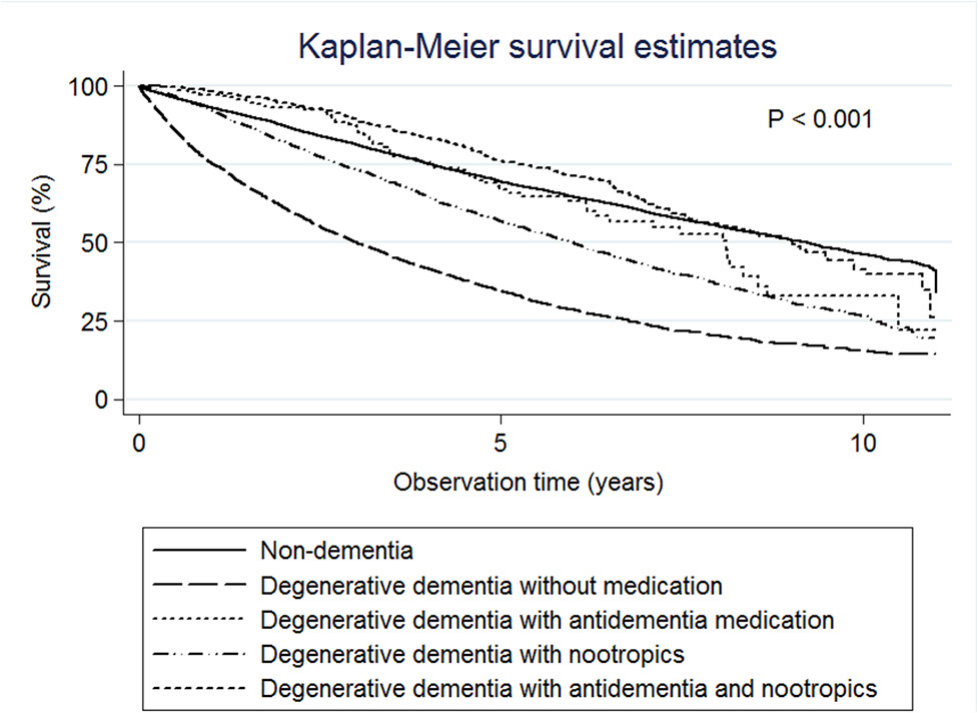
Kaplan-Meier survival estimates. The median survival times were 9.23 years for the non-dementia group, 3.01 years for degenerative dementia without medications, 8.11 years for degenerative dementia with anti-dementia medications, 6.00 years for degenerative dementia with nootropics, and 9.03 years for degenerative dementia with both nootropic and anti-dementia medications.

**Fig 3:**
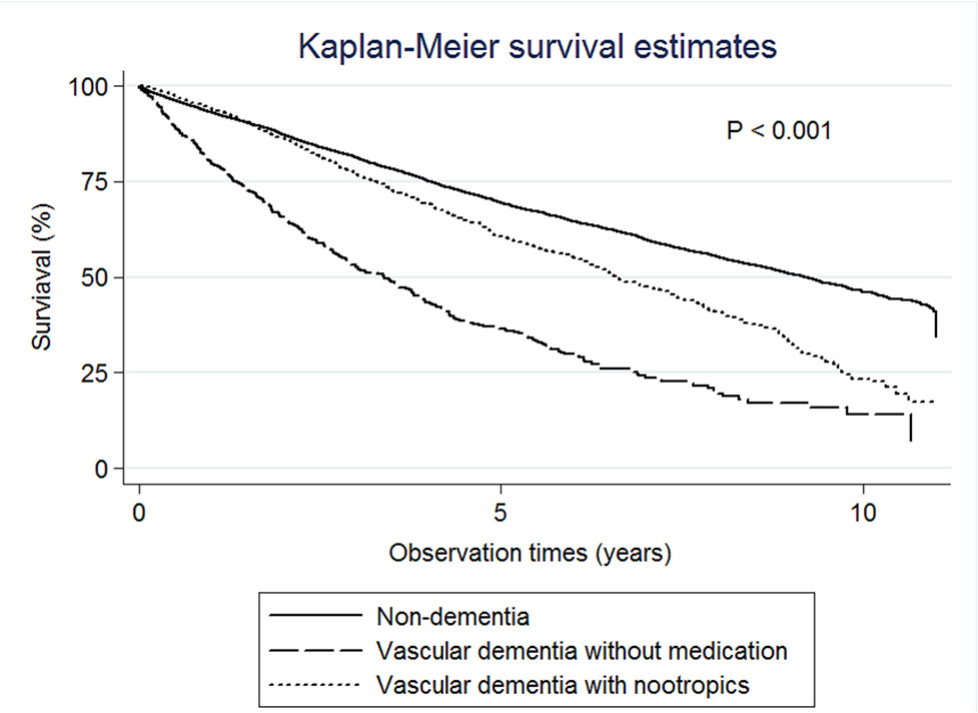
Kaplan-Meier survival estimates. The median survival times were 9.23 years for the non-dementia group, 3.39 years for vascular dementia without medications, and 6.62 years for vascular dementia with nootropics.

The median survival times were 3.01 years (95% CI: 2.85–3.21) for degenerative dementia without medications, 8.11 years (95% CI: 6.30–8.55) for degenerative dementia with anti-dementia medications, 6.00 years (95% CI: 5.73–6.17) for degenerative dementia with nootropics, and 9.03 years (95% CI: 8.02–9.87) for degenerative dementia with both nootropic and anti-dementia medications (Fig 2). The median survival times were 3.39 years (95% CI: 2.88–3.79) for VaD without medications, and 6.62 years (95% CI: 6.24–7.21) for VaD with nootropics (Fig 3).

### HRs for dementia with and without medications

The results of the survival models are shown in Table 4. The HRs were attenuated slightly when adjusted from the basic model to the full model. In the full model, the HRs of degenerative dementia were 2.69 (95% CI: 2.55–2.83) for those not using medications, 1.46 (95% CI: 1.39–1.54) for those using nootropics, 1.05 for (95% CI: 0.82–1.34) for those using anti-dementia medications, and 0.92 (95% CI: 0.80–1.05) for those using both nootropic and anti-dementia medications. The VaD cases that used nootropics had a lower mortality (HR: 1.25, 95% CI: 1.15–1.37) than those who were not using any medications (HR: 2.46, 95% CI: 2.22–2.72).

**Table 4:** Hazard ratios in the vascular dementia and degenerative dementia groups.

Variables	Basic model		Full model	
	HR	95% CI	HR	95% CI
Age (increased 1 year)	1.08	1.07–1.08	1.08	1.07–1.08
Male (ref: female)	1.36	1.30–1.41	1.29	1.24–1.34
Vascular dementia (ref: non-dementia)				
No medication	2.90	2.62–3.21	2.46	2.22–2.72
Nootropics	1.50	1.37–1.63	1.25	1.15–1.37
Degenerative dementia (ref: non-dementia)				
No medication	2.91	2.76–3.6	2.69	2.55–2.83
Nootropics	1.61	1.53–1.70	1.46	1.39–1.54
Anti-dementia medication	1.06	0.83–1.36	1.05	0.82–1.34
Both medications	0.96	0.84–1.10	0.92	0.80–1.05
Socioeconomic status (ref: low)				
Medium			0.99	0.92–1.06
High			0.98	0.92–1.04
Urbanity (ref: urban)				
Suburban			1.10	1.05–1.15
Rural			1.19	1.13–1.27
Charlson comorbidity index (increased 1)			1.14	1.13–1.14

## Discussion

Our study is unique because it utilized the general population, which is more representative of actual cases. Additionally, we used incident cases of dementia to evaluate the effects of anti-dementia and nootropic medications on patient survival.

Anti-dementia medications were introduced in the mid-1990s to treat AD [17]. Persistent anti-dementia medications are associated with a slower decline in cognition, even in patients with advanced diseases [11, 18, 19]. These mediations also significantly altered the treatment history by extending the time to nursing home admission [20, 21]. However, the effects of anti-dementia medications on longevity remain controversial [22]. Some studies found no relationship between anti-dementia medications and survival [23–25], while others reported a better survival rate for those using such medications [26, 27]. Nootropics are drugs used to treat cognition deficits in patients with AD, schizophrenia, stroke, and attention deficit hyperactivity disorder as well as aging patients and have also been use to improved cognitive impairment in patients with VaD. [13, 14, 28] The beneficial effects of nootropics on dementia are reportedly inconsistent and unreliable [13, 14]. Although anti-dementia drugs such as donepezil and memantine are used in clinical practice for treating VaD, they are not approved by the Food and Drug Administration of Taiwan and are not reimbursed for in the insurance policy. Therefore, anti-dementia agents are rarely used in Taiwan and were not analyzed in our study. In this population-based study, we found that nootropics alone have beneficial effects on mortality in patients with degenerative dementia and VaD. Although some studies have reported that anti-dementia medications do not prolong survival in patients with AD [11, 23–25], we observed beneficial effects of anti-dementia medication on the survival of patients with degenerative dementia, a finding also observed consistently in some other studies [26, 27, 29–31]. In patients with degenerative dementia who received only anti-dementia medication or a combination of anti-dementia and nootropic medications, the mortality risk reduced to that in patients without dementia. Besides, the patients who received the combined medication tended to showed greater advantages, which reflects the synergistic effect of anti-dementia and nootropics on dementia. Piracetam, a nootropic, has been shown to have a neuroprotective effect when used during coronary bypass surgery [32], and ginko biloba showed efficacy comparable to that of anti-dementia medication in the treatment of Alzheimer’s type dementia [33]. A recent study indicated a considerable overlap between cerebrovascular disease and AD and suggests that both pathologies have an additive effect on cognitive decline [34], which may support the possibility of a synergistic effect of anti-dementia and nootropic medications. However, further studies are needed to investigate the possible synergic effects of anti-dementia and nootropic medications.

In the NHI claim data, the prevalence of dementia among those aged 65 or above was 2.5% (2,646/103,797) in the year 2000, and increased to 6.6% (6,951/104,761) in the year 2010. These results are quite close to those of previous epidemiological studies conducted in Taiwan, where the prevalence rate in individuals aged 65 and over was reportedly 2.0–3.7% in the 1990s [35, 36]. and increased to 8% in 2011–2012 [37]. Therefore, the measurement of dementia cases should be valid.

The relative risk of mortality for patients with VaD is reportedly greater than the risk in AD, although this difference was not statistically significant [7, 38, 39]. In contrast, other reports have shown that patients with AD have a worse prognosis than those with VaD [40, 41]. In this study, patients with VaD tended to have a lower risk and slightly longer median survival time than patients with degenerative dementia. However, patients with VaD had a shorter survival time between the ages of 65 and 69. The risk reversed in those aged 85 and older, where degenerative dementia had a shorter survival time.

Our study revealed that the survival time among patients with dementia depends more on the age of the patient, which is consistent with a previous study [42]. Age and comorbidity remain the significant factors to mortality. Concomitant morbidities may increase mortality in most dementia cases [6]. Since concomitant illnesses commonly exist in elderly persons, it is difficult to investigate the direct associations between dementia and lifespan. In this study, we adjusted the models for CCI, where the elevation of one CCI score increased the mortality risk by 14%. The HRs of dementia decreased as the comorbidities were adjusted for in the full model. However, the relationship between dementia and mortality might be a marker of a global decline in health. Dementia is a complex series of symptoms with multiple causes, making it similar to most late-life chronic diseases.

Age-based stereotypes may lead to the underuse of medication based on the conclusion that an older patient’s malady is chronic and less susceptible to intervention [43]. Many people consider senility or dementia to be normal or an expected consequence of aging [44]. However, the impact of dementia on public health is enormous and will continue to grow, especially in an aging society. Dementia is a common and lengthy illness; therefore, even small treatment effects could have a substantial impact over time. In this study, patients with degenerative dementia who were using anti-dementia and/or nootropic medications had a more desirable outcome than those who were not using such medications, supporting the early use of these medications in dementia patients.

The results of this research should be viewed in the light of several limitations. First, the administrative data are subject to possible coding errors and under- or over-coding problems. Given that claims data have low sensitivity and high specificity for dementia diagnoses [45], the misclassification of some patients with milder dementia as being non-demented would underestimate our findings and imply that they are more conservative than they really are. Second, this study might suffer from certain inherent limitations because of the use of administrative data, which lack information on dementia severity. Third, drug therapy is usually not assigned randomly, so other unmeasured factors may influence the observed relationship between drug use and outcome. Behavioral and psychological symptoms of dementia (BPSD), such as psychosis, agitation, dysphoria, apathy, and disinhibition, occur in a majority of patients with dementia. The possible use of antipsychotics may be involved in an increase in mortality in these patients [46–48]. In this study, we focused on investigating the therapeutic effects of anti-dementia and nootropic medications on dementia-related mortality. Medication used for controlling BPSD was not investigated in our study. However, dementia patients who are treated with anti-dementia and nootropic agents may be more likely to be treated with their BPSD by their physicians than those who do not receive any treatment for dementia. Therefore, use of antipsychotic agents was not likely to be a cause of increased mortality in the non-treated group, which makes our results more conservative. Fourth, it was not possible to classify degenerative dementia into subtypes because of the data constraints, thus there is notable heterogeneity within this group. Fifth, although our analysis attempted to control for the severity of comorbidities, it is possible that there are residual confounding factors due to medical illness. Finally, the overall patterns of association between dementia and mortality for our population also reflect the organization of care for older people with dementia in Taiwan. The experience in other settings may be different if the care for this population is organized differently.

## Conclusion

The strength of our study is that we described the mortality of a large, nationwide population-based cohort of older adults with incident dementia, which enabled us to evaluate the effects of anti-dementia and nootropic treatments on mortality. Families of participants newly diagnosed with dementia often ask the physicians how long people commonly live with their treatment conditions. Our data revealed the importance of early treatment of dementia. In particular, combination treatment with anti-dementia and nootropics may prolong the life of patients with dementia. It provide a balanced response based on data from representative population-based samples, and should be relevant to health care planning and policy making for the growing number of individuals with dementia.
